# Comparison of years of life lost to 1,565 suicides versus 10,650 COVID-19 deaths in 2020 in Sweden: four times more years of life lost per suicide than per COVID-19 death

**DOI:** 10.48101/ujms.v127.8533

**Published:** 2022-05-11

**Authors:** Rickard Ljung, Maria Grünewald, Anders Sundström, Lena Thunander Sundbom, Björn Zethelius

**Affiliations:** a The Swedish Medical Products Agency, Uppsala, Sweden; b Institute of Environmental Medicine, Karolinska Institutet, Stockholm, Sweden; c Department of Public Health/Geriatrics, Uppsala University, Uppsala, Sweden

**Keywords:** Life expectancy, SARS-CoV-2, suicide, psychiatric disorders, burden of disease, public health

## Abstract

**Background:**

The burden of disease from the severe acute respiratory syndrome coronavirus 2 (SARS-CoV-2) pandemic is large; however, suicide affects the population year after year. From a public health perspective, it is important to not neglect contributors to the total burden of disease. The aim of this paper is to compare years of life lost (YLL) to suicide with those lost to coronavirus disease 2019 (COVID-19).

**Methods:**

A nationwide cohort study in 2020, in Sweden. YLL was measured as the sex- and age-specific remaining life expectancy at the time of the person’s death based on the death risks that pertained to the Swedish population in 2019. YLL to suicide was compared to YLL to COVID-19 and presented by sex and age groups. Suicide deaths in 2020 were estimated as the annual average of suicides in 2015–2019.

**Results:**

Annual average of suicide was 1,565, whereof 1,076 (68.8%) men and 489 (31.2%) women. In 2020, 10,650 persons died of COVID-19, whereof 5,681 (53.3%) men and 4,969 (46.7%) women. Estimated total YLL to suicide and COVID-19 in 2020 was 53,237 and 90,116, respectively. The COVID-19 YLL to suicide YLL ratio in 2020 was 1.69 (90,116/53,237). Men accounted for 67.1% of suicide YLL and of 56.4% of COVID-19 YLL. Those 44 years or younger accounted for 60.3% of suicide YLL and 3.9% of COVID-19 YLL. Those 75 years and older accounted for 2.9% of suicide YLL and 60.9% of COVID-19 YLL. On average, each suicide generates 34 YLL (53,237/1,565), and each COVID-19 death generates 8.5 YLL (90,116/10,650).

**Conclusions:**

YLL to suicide affects Sweden year after year, foremost attributable to the younger age groups, whereas YLL to COVID-19 is foremost attributable to the elderly. On average, each suicide generates four times more YLL than a COVID-19 death. Enormous efforts and resources have been put on tackling the pandemic, and without these, the burden would probably have been much larger. However, from a public health perspective, it is important to not neglect other contributors to the total burden of disease where national efforts also may have an impact.

## Introduction

The Global Burden of Diseases, Injuries, and Risk Factors (GBD) project provides a tool to quantify health loss to improve health systems and reduce inequalities in health (1). Psychiatric disorders and suicide are large contributors to the burden of disease foremost in the younger population (2–4). The contribution of communicable diseases to the leading causes of death has decreased in the last decades before the pandemic (2, 5). On 30 January 2020, the World Health Organization declared a Public Health Emergency of International Concern regarding coronavirus disease 2019 (COVID-19). Around 5.8 million confirmed attributed deaths, foremost among the elderly, are recorded as of January 2022 (6). The Swedish Medical Products Agency has, in the appropriation directions, received government assignments concerning psychiatric health and suicide prevention as well as COVID-19. Quantifying the burden of suicide relative to other diseases adds additional input to the agencies decision-making.

This study aimed to quantify the years of life lost (YLL) in 2020 in Sweden to suicide compared to YLL to COVID-19, by sex and age-groups and to discuss from a public health perspective the potential of joint national efforts.

## Methods

### Study design

This cohort study comprises all residents permanently residing in Sweden and alive on 31 December any year from 31 December 2014 to 31 December 2019 as recorded in the Total Population Register and was derived from the COVID-19 VACcination register SAFEty study in Sweden (CoVacSafe-SE) (7, 8). For the adult population, all deaths due to suicide or deaths with unclear intent in 2015–2019 and all deaths related to COVID-19 in 2020 were retrieved on individual level from the cause of death register (9). Suicide and COVID-19 deaths occurring in those younger than 18 years were retrieved in aggregated form.

### The Swedish cause of death register

The Swedish cause of death register contains information on personal identifier, sex, date of birth, date and underlying cause of death, and contributing causes of death for all deceased Swedish residents, including those occurring abroad, since 1952 and has a 99.2% completeness of causes of death (9).

### Register on surveillance of notifiable communicable diseases (SmiNet)

Information on notifiable diseases must be reported to SmiNet in accordance with Swedish law, the Communicable Diseases Act. SmiNet contains information on personal identifier, date of disease occurrence, date of testing, and date of positive test (10).

### Underlying cause of death

Death due to suicide was defined as an underlying cause of death with an International Statistical Classification of Diseases and Related Health Problems (ICD) code of suicide (X60-X84) or undetermined intent (Y10-Y34). Death due to COVID-19 was defined as an underlying cause of death of U071 (severe acute respiratory syndrome coronavirus 2 [SARS-CoV-2] identified) or U072 (clinical diagnosis of COVID-19), or any deaths occurring within 30 days of a positive COVID-19 test reported to SmiNet (9, 10).

### Years of life lost

For each deceased person due to suicide or COVID-19, YLL is the number of years lost due to premature death and is calculated as the remaining life expectancy at that specific age of death, representing the average number of years of life left for a hypothetical group of persons whom at that specific age were to live through their entire remaining lifespan with the same death risks that existed in Sweden in 2019 (11–13). Suicide in 2020 was estimated by using the annual average number of suicides by sex and age in 2015–2019 to account for time trend variation and delay in reporting. Suicide rates in Sweden have been stable during the last decade.

## Results

**Figure 1:**
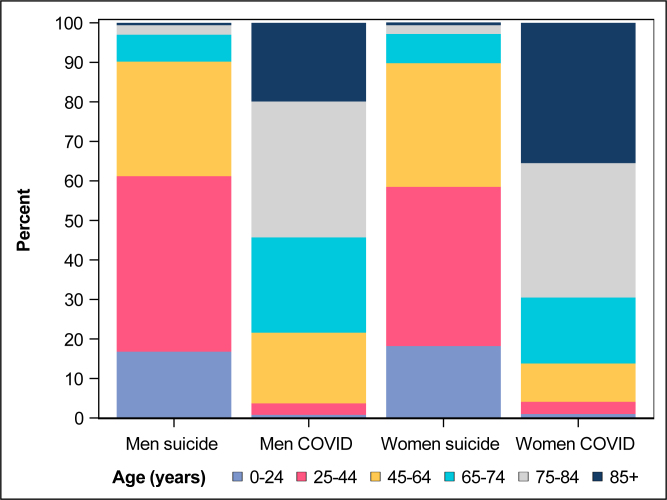
Proportional contribution of each age group to the total years of life lost (YLL) to suicide and to COVID-19 in 2020 in Sweden for men and women separately.

Annual average number of suicides in 2015–2019 was 1,565, whereof 1,076 (68.8%) men and 489 (31.2%) women. In 2020, 10,650 persons died of COVID-19, whereof 5,681 (53.3%) men and 4,969 (46.7%) women. Total YLL to suicide and COVID-19 in 2020 was estimated to 53,237 and 90,116, respectively (Table 1). The COVID-19 deaths to suicide deaths ratio was 6.8 (10,650/1,565). The COVID-19 YLL to suicide YLL ratio was 1.69 (901,16/53,237). Men accounted for 67.1% of suicide YLL and of 56.4% of COVID-19 YLL. Those 44 years or younger accounted for 60.3% of suicide YLL and 3.9% of COVID-19 YLL (Figure 1). Those 75 years and older accounted for 2.9% of suicide YLL and 60.9% of COVID-19 YLL.

**Table 1:** Number of suicides, as 5-year annual average, and deaths due to COVID-19 in 2020 in the Swedish population, and years of life lost (YLL) by sex and age. YLL derived from sex- and age-specific remaining life expectancy in 2019 in Sweden.*

	Suicide				COVID-19			
	*N*	%	YLL	%	*N*	%	YLL	%
Men	1,076		35,743		5,681		50,798	
00–24	98	9.1	5,999	16.8	6	0.1	395	0.8
25–44	331	30.7	15,870	44.4	32	0.6	1,463	2.9
45–64	361	33.5	10,376	29.0	350	6.2	9,087	17.9
65–74	152	14.1	2,445	6.8	800	14.1	12,243	24.1
75–84	91	8.5	856	2.4	2,000	35.2	17,484	34.4
85+	44	4.1	197	0.6	2,493	43.9	10,127	19.9
Women	489		17,494		4,969		39,318	
00–24	49	10.0	3,181	18.2	6	0.1	403	1.0
25–44	137	28.1	7,043	40.3	25	0.5	1,230	3.1
45–64	173	35.4	5,481	31.3	134	2.7	3,826	9.7
65–74	69	14.2	1,286	7.4	385	7.8	6,555	16.7
75–84	35	7.2	376	2.2	1,321	26.6	13,357	34.0
85+	25	5.1	126	0.7	3,098	62.4	13,946	35.5

* Remaining life expectancy at birth: 85 years for women and 81 years for men.

Of suicide deaths, 1,216 (77.7%) were from suicide and 349 (22.3%) from death with undetermined intent. Of COVID-19 deaths, 9,006 (84.6%) were from underlying cause of death U071, 428 (4.0%) from U072, and 1,211 (11.4%) from 30 days since positive COVID-19 test.

On average, each suicide generates 34 YLL (53,237/1,565), and each COVID-19 death generates 8.5 YLL (90,116/10,650).

## Discussion

This population-based nationwide study shows most of YLL to suicide was in those 44 years and younger, whereas most of YLL to COVID-19 was in the old and very old. YLL to COVID-19 in 2020 was around 70% higher than YLL to suicide. For both suicide and COVID-19, YLL was higher for men than for women.

The main strengths include the population-based cohort design with full nationwide coverage of the population and complete follow-up of deaths. We chose to present data on the maximal possible number of suicides by including both suicide and death with undetermined intent (14), and the maximal possible number of deaths related to COVID-19 by including all deaths with an underlying cause of death related to COVID-19 and death occurring within 30 days of a positive test for COVID-19, as proposed by the Public Health Agency of Sweden. These choices could be an over-estimation of both suicides and COVID-19 deaths.

In line with the current recommendations from GBD, we applied neither age-weighting nor discounting of YLL (15). Age-weighting, where YLL in children or the elderly is counted less, would have reduced the YLL in COVID-19 more than in suicide. This would have reduced the relative difference between the two conditions. Discounting where future YLL is discounted would probably have reduced the YLL to suicide more than to COVID-19 as most of the YLL in suicide lays far in the future compared to YLL to COVID-19 where the remaining life expectancy in the elderly deceased from COVID-19 lays only a few years ahead. As a more interpretable and simplistic approach, we used the actual sex-specific remaining life expectancy in Sweden in 2019 to calculate YLL, with a sex gap of around 4 years in favor of women (remaining life expectancy at birth of 85 years for women and 81 years for men) (2, 5, 15). If we would have used the global burden of disease reference life table for 2010 with a life expectancy at birth of 86 years for both sexes, this would increase YLL to both suicide and COVID-19 relatively more for men than for women (2, 15).

Between country comparisons of YLL can be informative but the calculation of disease specific YLL can be prone to country specific differences in health care system and resources, and quality of cause of death statistics, as well as to diagnostic procedures and testing as can be seen in between country comparisons of YLL to COVID-19 (16). Without competing risk of suicide persons with psychiatric disease still have a shorter life expectancy (14, 17). Similarly, YLL to COVID-19 is mostly attributable to the fragile comorbid old population (18).

Regardless of the methodological considerations above, YLL to suicide is more than half of YLL to COVID-19 in 2020. Enormous efforts and resources have been put on tackling the pandemic, and without these, the burden would probably have been much larger. However, the pandemic has shown that countries can vigorously pursue necessary action for improving public health. YLL to suicide affects Sweden repeatedly, ongoing year after year. Hence, extrapolating the total burden of suicide in the last 10 years compared to COVID-19 in 2020 yields that more than five times of YLL are lost to suicide than to COVID-19 in the last decade. The Swedish Medical Products Agency participates in a joint effort of several government agencies to develop a national strategy for psychiatric health and suicide prevention, initiated by the Swedish government in 2020. The Agency also participates in the assessment of marketing authorization applications as well as the monitoring of safety of COVID-19 vaccines within the EU. Evaluating the number of potential YLL to suicide and to COVID-19 could from a public health perspective be informative for actions and decisions of government agencies.

## Conclusion

YLL to COVID-19 in 2020 was around 70% higher than YLL to suicide despite that COVID-19 deaths were more than six times more common than suicide. Hence, a suicide generates four times more YLL than a COVID-19 death. From a public health perspective, it is important to not neglect diseases that contribute to the total burden of disease, ongoing year after year and where joint national efforts may have an impact.

## Data Availability

According to Swedish Law, the data cannot be placed in a publicly available repository. Researchers can, after ethical approval from the Swedish Ethical Review Authority (www.etikprovningsmyndigheten.se), apply for data from the National Board of Health and Welfare, Stockholm, Sweden (www.socialstyrelsen.se). The Swedish Medical Products Agency will consider proposals for research collaboration. Enquiries can be submitted to the agency (registrator@lakemedelsverket.se with a copy to the corresponding author rickard.ljung@lakemedelsverket.se).
